# Mellitic Acid as a Stable Abiotic Precursor for Liquid‐Liquid Phase Separation: An RNA‐Independent Pathway for Prebiotic Compartmentalization

**DOI:** 10.1002/chem.202501526

**Published:** 2025-08-13

**Authors:** Robert Dec, Michel W Jaworek, Matylda Wacławska, Wojciech Dzwolak, Roland Winter

**Affiliations:** ^1^ Department of Chemistry and Chemical Biology Physical Chemistry I – Biophysical Chemistry TU Dortmund University Otto‐Hahn Street 4a 44227 Dortmund Germany; ^2^ Faculty of Chemistry Biological and Chemical Research Centre University of Warsaw Pasteur Street 1 Warsaw 02–093 Poland

**Keywords:** liquid‐liquid phase separation, membraneless organelles, protocells

## Abstract

Apart from playing the well‐recognized roles in the self‐assembly of intracellular machinery liquid‐liquid phase separation (LLPS) constitutes a hypothetical, yet compelling scenario for prebiotic compartmentalization. One challenge facing this idea is the absence (or scarcity) of typical biopolymer precursors of liquid droplets, such as RNA, on early Earth. We demonstrate that mellitic acid (MAc), an abiotic component found in certain minerals and a marker of organic matter in astrobiology, forms liquid droplets with poly‐L‐lysine (PLL) across a broad pH range and various mixing ratios. The chain‐length threshold of PLL capable of forming droplets with MAc ((L‐Lys)_6_) is significantly lower than when ATP, the primary intracellular energy currency, is used to induce LLPS in PLL. MAc‐PLL droplets are highly dynamic systems that respond reversibly to added ATP through an efficient mixing and demixing behavior. The observed effects of pH, ionic strength, temperature, and pressure, as well as isothermal titration calorimetry (ITC) data, are all consistent with coulombic interactions being the key contributor to maintaining the droplet state in the MAc‐PLL system. We propose that the capacity to induce LLPS with short oligocations, along with abiotic synthesis routes, positions mellitic acid as a viable candidate for an ingredient of early prebiotic membraneless protocells.

## Graphical abstract

Evidence is presented that prebiotically accessible mellitic (benzenehexacarboxylic) acid (MAc) is forming liquid droplets with short poly‐L‐lysine. The capacity of MAc to induce liquid‐liquid phase separation with short oligocations, along with abiotic synthesis routes, makes MAc a viable candidate for an ingredient of early prebiotic membraneless organelles.

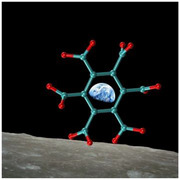

## 1 Introduction

The phenomenon of liquid‐liquid phase separation (LLPS) has been linked to the self‐assembly of various membraneless organelles, such as stress and germ granules.^[^ ^1^, ^2^, ^3^, ^4^ ^]^ Formation of these intracellular liquid droplets through the condensation of proteins (and nucleic acids) provides microenvironments with distinct solute concentrations, biopolymer dynamics, viscosity, pH, and polarity, factors that benefit various processes run by the biomolecular machinery of the cell.^[^ ^5^ ^]^ On the other hand, LLPS has also been implicated in protein misfolding and its disease‐related dysfunction.^[^ ^6^, ^7^, ^8^, ^9^ ^]^ Currently, the pathogenic interplay between LLPS and amyloidogenesis is an intensively researched topic within biophysics and molecular medicine.^[^ ^10^ ^]^

Apart from these two main aspects through which LLPS is regarded in the field of “mature” biological systems, another significant consideration involves LLPS's potential role in the emergence of life on Earth.^[^ ^11^, ^12^, ^13^ ^]^ Microscopic compartmentalization that maintains chemical potential gradients while overcoming entropic barriers faced by higher‐order reactions in solution was arguably one of the most essential requirements for early prebiotic self‐replicating systems. In light of the absence of membrane‐forming phospholipids on prebiotic Earth, formation of liquid droplets through LLPS has become a compelling idea for how this compartmentalization was achieved – a notion famously articulated by Oparin as early as the 1920s.^[^ ^14^ ^]^ Consequently, it is not surprising that research efforts are increasingly focused on identifying simple compounds capable of undergoing LLPS in aqueous environments deemed relevant to prebiotic conditions.^[^ ^15^ ^]^

**Figure 1:**
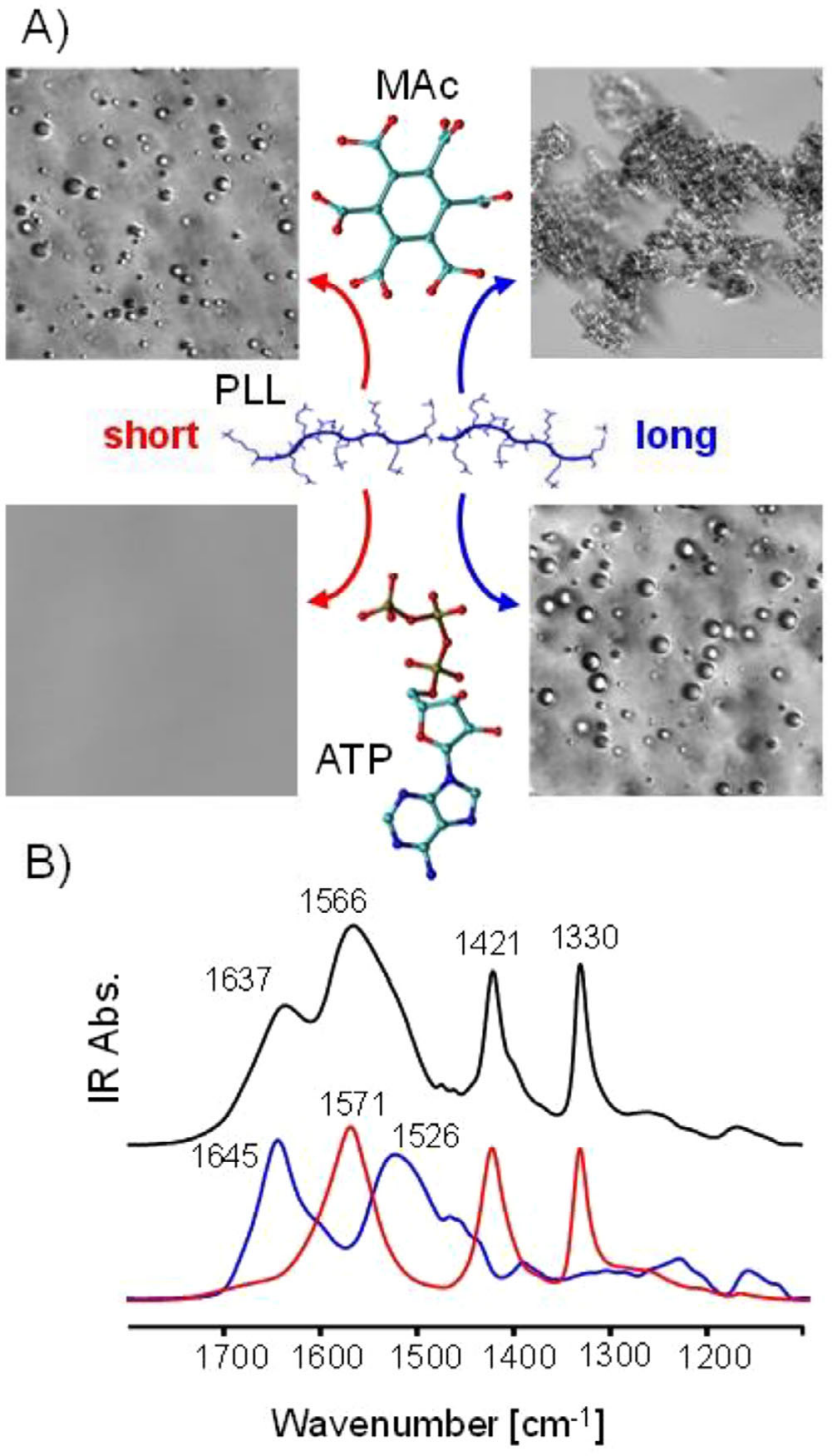
LLPS in the poly‐L‐lysine (PLL) + mellitic acid (MAc) system. Microscopic images of liquid droplets induced in aqueous PLL by MAc and ATP. Short PLL (1‐5 kDa) forms droplets with MAc while ATP‐based LLPS requires longer PLL (15‐30 kDa). All samples were 17.9 mM per lysine (K) residue; the molar mixing ratio was 1:6 (MAc:K) or 1:3 (ATP:K), pH 6.5, images collected 5 minutes after the mixing under ambient conditions A). FT‐IR spectra of dried films obtained from the dense droplet phase of the MAc‐PLL_SHORT_ system (black line at the top) and nonmixed solutions of PLL (blue line) and MAc (red line) both pH‐preadjusted to 6.5 B).

The highly symmetric structure of mellitic (or benzenehexacarboxylic) acid (Figure 1) contributes to its stability under various environmental conditions, while the mutual proximity of carboxyl groups results in a distinctive dissociation pattern characterized by a remarkable spread of pK_a_ values, ranging from roughly 0.7 to 7.5.^[^ ^16^, ^17^ ^]^ The symmetry and the abundance of densely packed carboxyl groups, which can engage in hydrogen bonding and salt bridge formation depending on pH, render mellitic acid a versatile building block for self‐assembled molecular networks, such as metal‐organic frameworks.^[^ ^18^, ^19^ ^]^ Importantly, mellitic acid may be obtained through entirely abiotic routes – for example as an intermediate product of oxidation of graphite in a strictly inorganic environment.^[^ ^20^, ^21^ ^]^ The aluminum salt of mellitic acid (Al_2_[C_6_(COO)_6_]·16 H_2_O) occurs in nature as *mellite* – a crystalline mineral unique in having an aromatic system as a building block.^[^ ^22^ ^]^ So far, the fact that MAc can be formed abiotically from carbon has not translated into specific mechanistic paradigms in the field of prebiotic chemistry. However, the nonvolatile character and overall stability of mellitic acid, along with its recognition as a persistent remnant of incompletely oxidized (bio)organic matter, have sparked considerable interest regarding its role as a reporter molecule in astrochemistry and astrobiology.^[^ ^23^, ^24^, ^25^, ^26^ ^]^ In fact, dedicated analytical approaches are being developed with the sole purpose to adequately detect MAc in the Martian regolith (e.g.,^[^ ^24^ ^]^) while oxidation of polycyclic aromatics to MAc under laboratory conditions is used to quantify organic matter present in lunar regolith.^[^ ^27^ ^]^

Strongly charged homopolypeptides, such as poly‐L‐lysine (PLL), poly‐L‐arginine, or poly‐L‐glutamic acid, are often matched with proper counterions (e.g., ATP for PLL) to form droplets serving as model biomimetic protocells.^[^ ^28^ ^]^ In the seminal work by Koga et al., aqueous solutions of ATP and PLL (of sufficient chain lengths) have been used to form microdroplets.^[^ ^29^ ^]^ The chain length of these homopolypeptide precursors is one of the key factors determining the phase behavior and the conformational dynamics within the coacervates.^[^ ^30^, ^31^ ^]^ Intuitively, the biopolymer chains must reach a sufficient length for LLPS to take place, although very long chains tend to form solid aggregates instead of liquid droplets. Hence, relatively short oligo‐ion species may outperform their longer variants in the capacity to form droplets stable over experimentally accessible timescales.^[^ ^32^ ^]^ This observation also resonates with the justification of exploring droplet building blocks among shorter peptides since very long homopolypeptides are clearly less likely to form abiotically. In fact, identifying simple stable molecules that are both abiotically accessible and potentially abundant in local environmental niches as plausible candidates to form liquid droplets remains one of the key challenges to the hypothesis that LLPS drives early protocell compartmentalization. Here, with our newly presented evidence demonstrating that MAc forms liquid droplets with very short polylysine chains, we strongly advocate for consideration of an involvement of MAc in the formation of prebiotic coacervates.

## 2 Results and Discussion

In selecting MAc as a potential candidate molecule promoting liquid droplet formation we considered the extreme charge densities on MAc^5‐^ and MAc^6−^ anions capable of triggering rapid self‐association of positively charged globular proteins.^[^ ^33^ ^]^ For initial tests, two commercially available types of PLL (labelled as PLL_SHORT_ and PLL_LONG_ with the corresponding approximate molecular weights 1–5 and 15–30 kDa, respectively–Supporting Information) were used. Preliminary experiments were carried out at near‐neutral pH and at the MAc‐PLL mixing ratio corresponding to the exact match of negative (on MAc) and positive (on PLL side chains) charges, assuming a complete dissociation of carboxyl groups and a full protonation of lysine side chains. The results are juxtaposed to those obtained for the ATP‐PLL pair first described by Koga et al.^[^ ^29^ ^]^ in Figure 1A. The key positive outcome of this experiment, that is, the straightforward microscopic evidence of liquid droplets formed upon mixing of MAc with short PLL, became the starting point prompting a more detailed investigation. Importantly, MAc induces LLPS involving polydisperse PLL_SHORT_ chains (8–40 residues), which are too short to form liquid droplets with ATP. Only when the significantly larger (and arguably less prebiotically accessible) PLL_LONG_ (approximately 110–220 residues long) is used, droplets appear in the ATP‐based system. We note that MAc forms solid aggregates with PLL_LONG_ at the same pH, which is not unexpected in light of earlier works on the impact of polyions of different sizes on LLPS systems.^[^ ^28^, ^32^ ^]^

The infrared spectrum of the centrifuged dense MAc‐PLL_SHORT_ droplet phase is shown in Figure 1B along with the control spectra of separate MAc and PLL_SHORT_ collected prior to the mixing. A broad band around 1637 cm^−1^ stemming from the amide I band overlapped with the MAc ring vibration is flanked by a larger peak at 1566 cm^−1^, which is due to the superposition of the amide II band with mostly antisymmetric stretches of carboxyl groups. The amide I band's large bandwidth points to the lack of ordered secondary structures. We have attempted to probe the polypeptide's conformation in MAc‐PLL_SHORT_ droplets with complementary far‐UV CD spectroscopy (Figure S1). However, despite employing an extremely short optical path of 0.01 mm, the centrifuged droplet phase was still too optically dense to allow acquisition of high‐quality data. We note, that in‐droplet MAc anions are unlikely to become arranged in chiral staircase‐like superstructures, as this would result in ππ* transitions below 280 nm becoming CD‐active. In the partly accessible range of amide nπ* and ππ* transitions below 230 nm, the spectrum is flat, which in agreement with the IR data, is indicative of structural disorder. This contrasts with the reported propensity of PLL to acquire an α‐helical conformation in ATP‐triggered droplets.^[^ ^29^ ^]^ Our previous works on chimeric peptides with merged amyloidogenic segments of insulin (ACC_1‐13_) and PLL (K_n_) chains of different lengths^[^ ^34^, ^35^ ^]^ revealed that triggering multistage LLPS → amyloid processes by binding and incorporating ATP molecules is accompanied by the electronic transitions within the adenine moiety becoming CD‐active, that is, chiral superstructures of ATP are formed.^[^ ^36^ ^]^ Arguably, in the case of MAc‐PLL_SHORT_ droplets, π‐π stacking interactions could be hindered by strong coulombic repulsion between the MAc anions, although those, along with Lys^+^ cation – MAc π‐electron‐system interactions could also contribute to the stability of the droplet state. Over prolonged ambient‐conditions incubation, freshly formed tiny MAc‐PLL_SHORT_ droplets merge into larger entities, as is expected for a coacervate system (Figure S2). We also note that MAc‐PLL_SHORT_ coacervates destroyed through drying readily reconstitute the liquid droplet state upon rehydration (Figure S3)–a minor fact that is of some interest from the perspective of possible instabilities in prebiotic environments.

**Figure 2:**
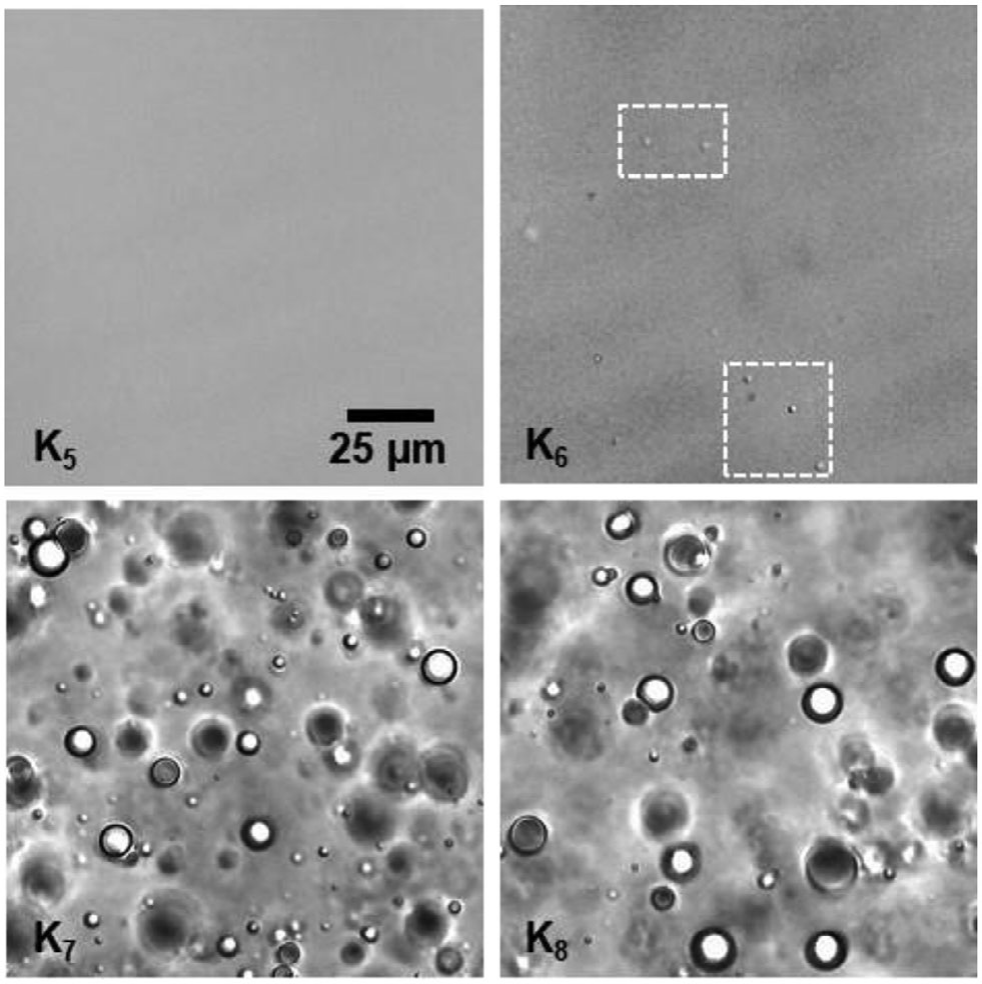
Impact of the degree of polymerization of PLL on LLPS in the presence of MAc. Oligo‐L‐lysine peptides were used at the uniform 17.9 mM per K residue concentration; MAc was present at 1:6 MAc:K molar ratio, pH 6.5, room temperature (20 °C). Tiny droplets appearing in the MAc‐K_6_ system are highlighted in frames.

Next, we used a series of custom‐synthesized oligolysine peptides with a controlled number of residues to determine the exact molecular weight cut‐off below which LLPS does not occur. According to the microscopic data presented in Figure 2, K_6_ is the shortest oligolysine peptide still capable of forming droplets with MAc (mixing stoichiometry 1MAc:6 K) at pH 6.5, with the resulting droplets being finer and less abundant than those formed by K_7_ and K_8_. The fact that MAc is capable of pairing with such low‐molecular‐weight homooligopeptide to form droplets is significant from the standpoint of the plausible prebiotic scarcity of larger macromolecules. Importantly, LLPS may be favored by a variety of physicochemical parameters not explored here (higher total concentrations, different pH, lower temperature, different osmotic or hydrostatic pressure, macromolecular crowding,^[^ ^37^, ^38^ ^]^ etc.). When acting synergistically, the cut‐off “n”‐value for LLPS to take place in MAc‐K_n_ systems may be even lower than 6.

**Figure 3:**
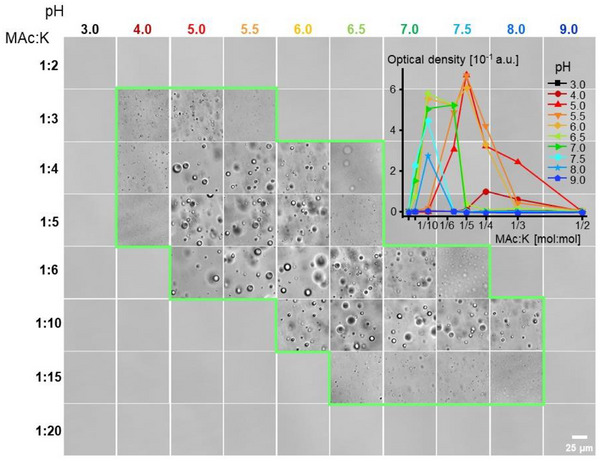
Dependence of LLPS in the MAc‐PLL_SHORT_ system on the pH and mixing ratio as probed by optical microscopy – a tentative phase diagram. The approximate demixing area is demarcated with the green line. Corresponding quantitative measurements of the optical density (at 500 nm, 3 mm long quartz cuvette) are presented in the inset; other conditions: 17.9 mM per K residue of PLL, room temperature (20 °C).

The stability of liquid droplets is simultaneously determined by various factors, making the mapping of multidimensional LLPS phase diagrams an exceptionally laborious task. In this study, we have focused on two essential factors, namely: pH and the mixing ratio. Figure 3 presents an approximate phase diagram of the MAc‐PLL_SHORT_ droplet state in relation to pH and mixing ratio. The microscopic and optical density measurements were conducted under ambient temperature and pressure conditions. The droplet area expands over 5 pH units (from 4 to 8), with the corresponding molar MAc:K mixing ratio ranging from approximately 1:3 to 1:15. Within this pH range, most of PLL's side chains are protonated. Hence, the changing pH affects the system by shifting equilibria between various mellitate anions. The less deprotonated MAc^m−^ species are saturated with fewer Lys^+^ cations, as reflected in the decreasing MAc:K mixing ratio in acidified samples. We observed a notable polymorphism among the droplets formed under different conditions: those in the central area of the diagram (i.e., at pH close to neutral and the mixing ratio close to 1:6) are larger than the tiny specimens dominating in the vicinity near the borderline of the phase coexistence curve. The effect could be kinetic in nature, that is, tiny droplets formed at more extreme pH values may require more time to merge into larger coacervate bodies due to the electrostatic repulsion between droplet interfaces in this metastability region. It is important to emphasize that these different types of droplet morphologies correspond to different concentrations of ‐COO^−^ – Lys^+^ bridges (which increase with pH), providing locally co‐dissolved ionic species with varying degrees of Debye screening.

**Figure 4:**
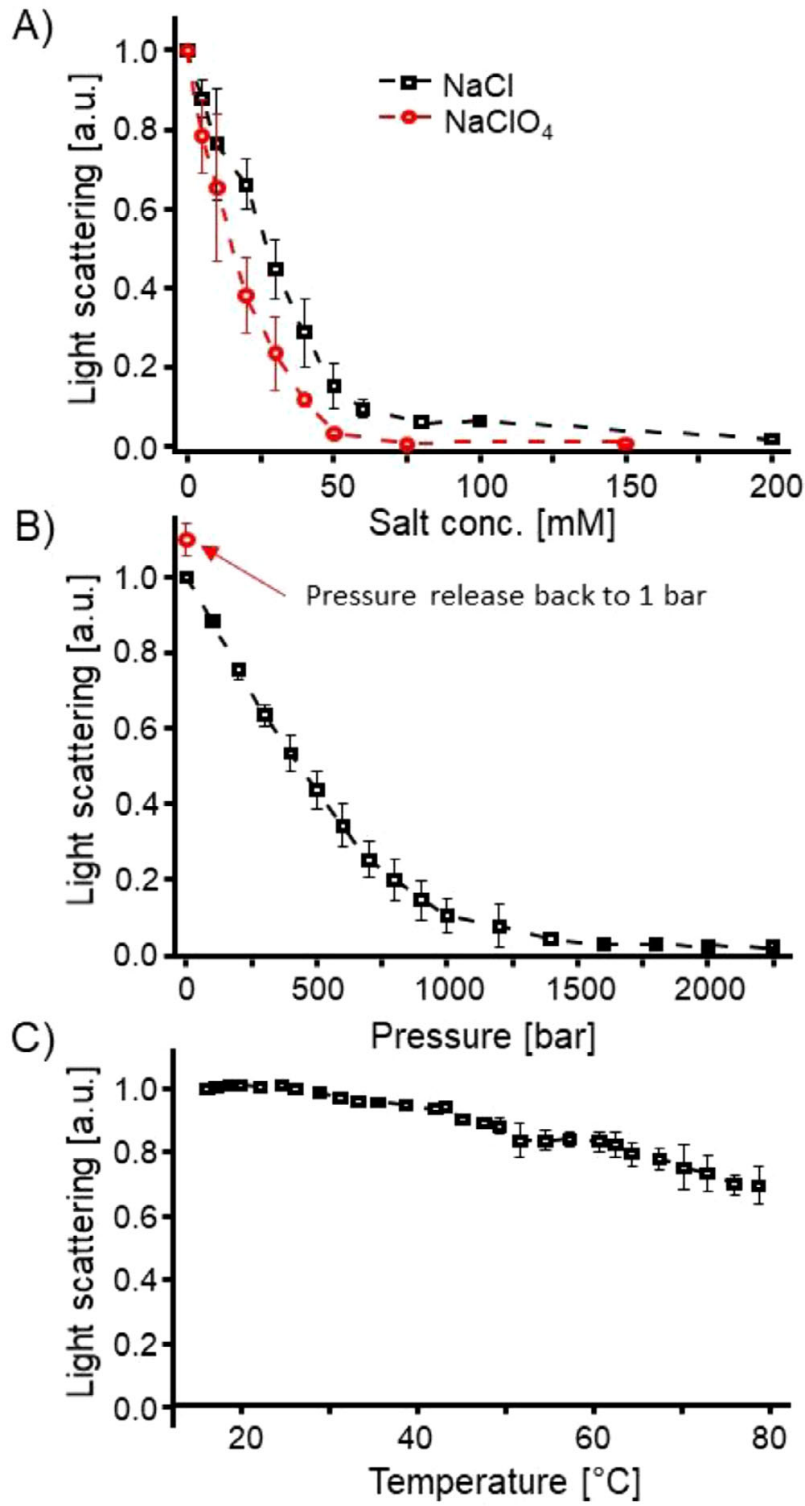
Stability of MAc‐PLL_SHORT_ liquid droplets against ionic strength A), hydrostatic pressure B) and temperature C), probed by static light scattering (normalized) at ∼400 nm. Sample conditions: PLL conc. of 12 mM per K residue, MAc:K molar ratio 1:6, pH 6.5, 20 °C.

Subsequently, high salt concentrations (Figure 4A), high hydrostatic pressure (Figure 4B), and high temperature (Figure 4C) were employed to probe the stability of the MAc‐PLL_SHORT_ phase preformed at typical pH/mixing ratio values. Since chaotropic perchlorate salts are present at significant concentrations in the Martial soil,^[^ ^39^, ^40^, ^41^ ^]^ these salts have been used to probe the stability of liquid condensates as well as RNA and biomembranes in an astrobiological context investigating the potential habitability of the Martian subsurface.^[^ ^42^, ^43^, ^44^, ^45^, ^46^ ^]^ Along with NaCl, we used NaClO_4_ here to study the impact of ionic strength on MAc‐PLL_SHORT_ droplets. As expected for a system stabilized by dynamic networks of mostly electrostatic interactions, increasing ionic strength reverses the phase separation propensity of MAc‐PLL_SHORT_. We note that both salts act similarly in this respect, although NaClO_4_ appears to be more effective. In principle, the observed susceptibility of MAc‐PLL_SHORT_ droplets to high ionic strength should be mirrored in the system's sensitivity to high hydrostatic pressure, as polar water molecules pack more tightly around dissociated than intact salt bridges due to the so‐called electrostriction phenomenon. We have confirmed this through high‐pressure measurements of optical density, that is, through turbidity measurements (Figure 4B). The observed pressure‐induced dissociation of the droplets is fully reversible. It should be stressed that the complete mixing is observed only above 1000 bar, that is, under the hydrostatic pressure of extreme oceanic depths of 10,000 m, while most of the deep‐sea hydrothermal vents considered in the prebiotic context on Earth are 2000–3000 m below sea level with corresponding hydrostatic pressures on the order of 200–300 bar.^[^ ^46^, ^47^ ^]^ The high pressure‐induced instability of droplets contrasts with their relative durability at increasing temperatures (Figure 4C), implying that hydrogen bonds and hydrophobic interactions between nonpolar groups are not critical for maintaining the stability of the droplet state. While an interplay of multiple noncovalent interactions, including hydrogen bonding, π‐π stacking, hydrophobic effects, and coulombic interactions typically governs stability of the droplet state in biomacromolecular systems,^[^ ^48^, ^49^, ^50^ ^]^ the MAc‐PLL system appears to be particularly dependent on the electrostatics. This is consistent with the effects of pH, ionic strength, hydrostatic pressure, and high temperature. For example, according to the phase diagram in Figure 3, droplets are most abundant at pH favoring straightforward electrostatic interactions between MAc and PLL, rather than at low pH conditions, which could be more permissive to π‐π stacking (MAc‐MAc) or cation(Lys^+^)‐π(MAc)‐interactions. We have also employed isothermal titration calorimetry (ITC) to probe the thermodynamics of MAc‐PLL binding within droplets at pH 7.5 (Figure S4). The ITC‐derived MAc‐PLL_SHORT_ binding stoichiometry molar ratio of 0.14 (per K residue) is close to the value expected for a complete saturation of coulombic interactions between fully ionized carboxyl groups of MAc and fully protonated lysine side chains of PLL (0.166). The binding process (∆*G* = ‐37.8 kJ/mol) is exothermic (∆*H* = ‐21.4 kJ/mol), but is also entropy‐driven (*T*∆*S* = 16.4 kJ/mol). The negative ∆*H* value supports the intuitive idea that hydrophobic interactions are not the main driving force of the MAc‐PLL association while the positive ∆*S* value points to the desolvation of interacting surfaces occurring upon LLPS.^[^ ^51^ ^]^ Again, the ITC data appears to resonate with the earlier reported electrostriction‐driven high‐pressure‐induced dissolution of droplets.

**Figure 5:**
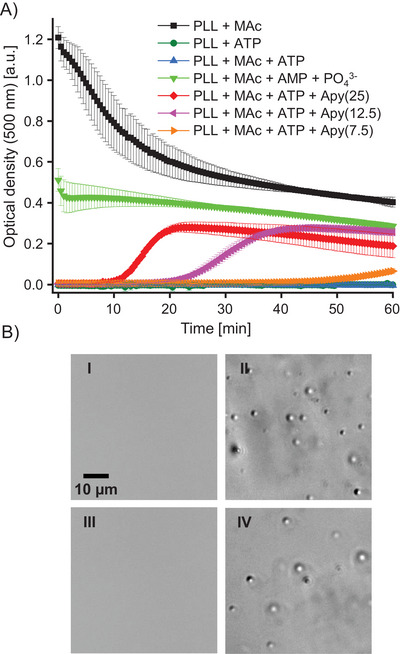
ATP prevents formation of droplets in the MAc‐PLL_SHORT_ system. Formation of droplets probed by time‐dependent optical density measurements at 500 nm in various mixtures containing PLL_SHORT_ (17.9 mM per K residue), MAc (1:6 MAc:K molar ratio), pH 6.5, 20 °C. ATP was added in 2.5‐fold stoichiometric excess (2.5 ATP molecules per 3 K residue). Apyrase (Apy) was added at the concentration specified [µg/mL]; all samples contained also 5 mM CaCl_2_ A). Optical microscopic images of PLL_SHORT _+ MAc + ATP (I), PLL_SHORT _+ MAc + AMP + PO_4_^3‐^ (II), freshly prepared PLL_SHORT _+ MAc + ATP + Apy at 5 µg/mL (III), and the same sample after 1 hour long incubation at 30 °C (IV) B).

**Figure 6:**
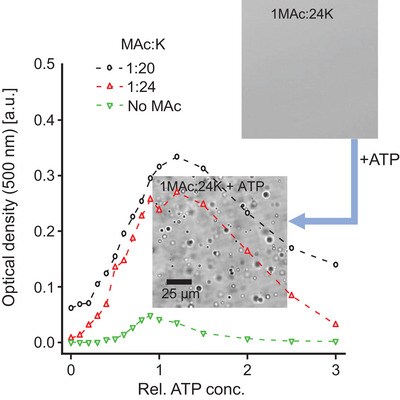
Titration of single‐phase MAc‐PLL_SHORT_ systems with ATP probed by optical density measurements at 500 nm. In both cases (MAc:K – 1:20, 1:24), the concentration of MAc is insufficient to form droplets. Addition of ATP triggers transient LLPS. The relative ATP conc. of 1 is defined as the ATP concentration required to substitute the missing portion of MAc needed to fully match charges on lysine side chains (assuming complete protonation of PLL side chains as well as complete deprotonation of MAc and the presence of ATP^3−^ ions, only). Other conditions: PLL_SHORT_ (17.9 mM per K residue), pH 6.5, 25 °C, measurements were conducted 5 minutes after mixing solutions of MAc and PLL_SHORT_. Microscopic images in the inset depict formation of liquid droplets upon addition of ATP (relative conc. = 1) to the MAc‐PLL_SHORT_ (1:24) single‐phase system.

An interesting observation concerns a mixed system in which MAc, ATP, and PLL_SHORT_ are simultaneously present, allowing one to probe the impact of ATP, the energy currency of the biological cell, on the system. As we stated earlier, unlike MAc, ATP (under the conditions of this study and when matched to a particular low‐molecular‐weight fraction of PLL) does not effectively induce liquid droplets. However, when an excess of ATP is added to MAc‐PLL_SHORT_ droplets, the whole system becomes a single liquid phase. Data presented in Figure 5 depict how the presence of a 2.5‐fold molar excess of ATP prevents LLPS in the MAc‐PLL_SHORT_ system. Clearly, the nucleotide competes for MAc interactions with PLL without condensing into ATP‐PLL_SHORT_ droplets. Should the ATP concentration be reduced, for example by adding an ATP‐hydrolyzing enzyme (apyrase), MAc‐PLL_SHORT_ droplets will reappear on the timescale corresponding to the kinetics of the enzymatic reaction (Figures 5A and S5), as the ultimate products of ATP hydrolysis (AMP and phosphate) are incapable of disrupting the droplets. This is reminiscent of how selectively ATP (unlike ADP, AMP, and phosphates) triggers LLPS in ACC_1‐13_K_n_ chimeric peptides described recently.^[^ ^34^ ^]^ The competitive removal of polylysine chains from MAc‐PLL_SHORT_ droplets by ATP has also been captured in another experiment reported in Figure 6. Here, ATP was gradually added to a single‐phase mixture of PLL_SHORT_ with a reduced amount of MAc (3–4 times below the optimum stoichiometric ratio), in which scarcity of thermal fluctuation‐damping ionic cross‐links prevents the system from undergoing LLPS. However, as the titration curve reveals, with the stepwise addition of ATP binding the excess of PLL, a balanced stoichiometry between MAc and the remaining peptide is restored allowing droplets to form. Once the ATP concentration crosses the optimum threshold (marked as “1”, corresponding to the amount required to saturate the unbound fraction of PLL), the nucleotide's competitive interactions with the peptide decrease the overall concentration of droplets.

**Figure 7:**
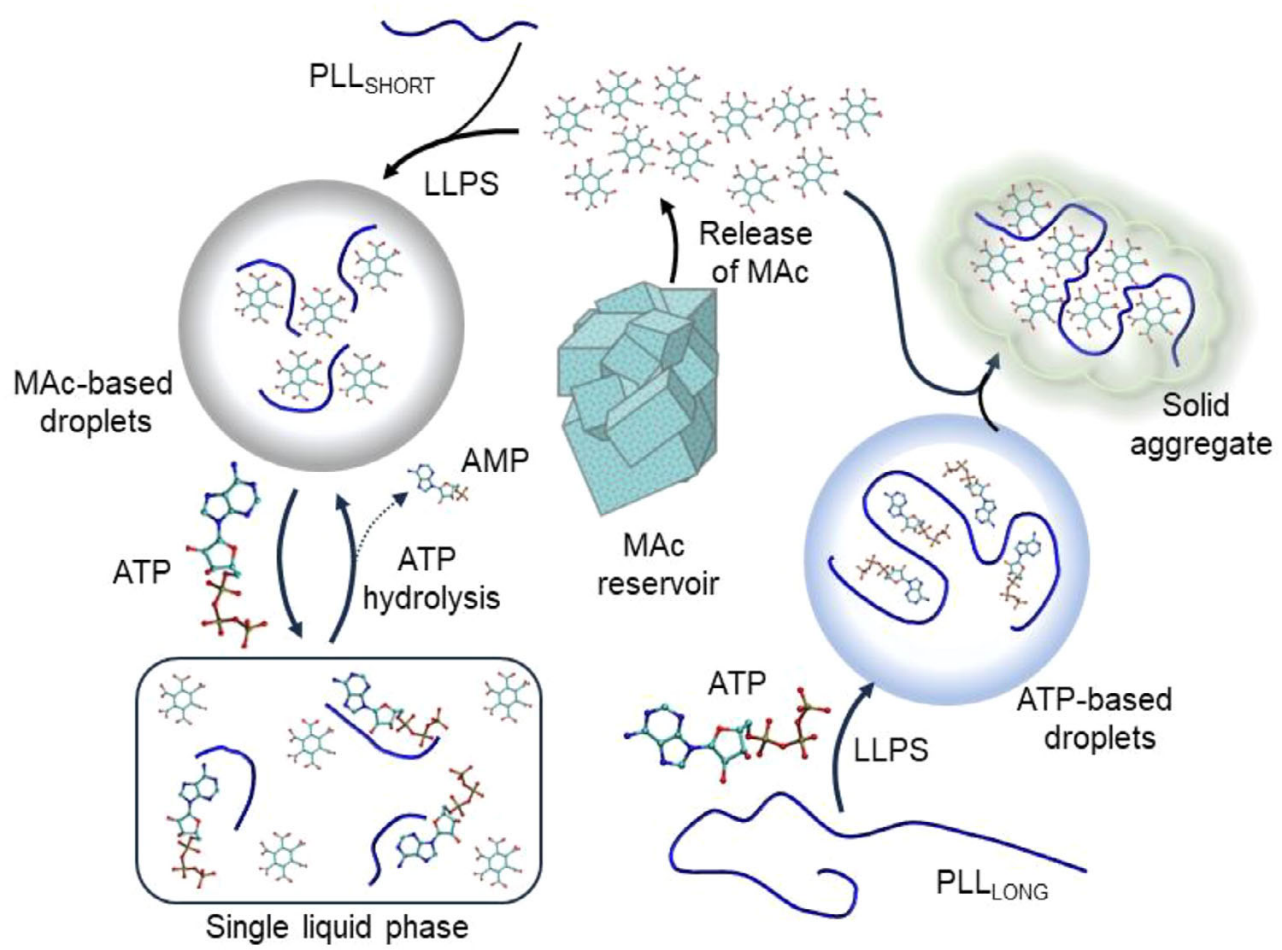
Schematic representation of the phase transitions involving mellitic acid studied in this work. In the prebiotic context, the reservoir of MAc could be mineral.

The fact that ATP outcompetes MAc from binding to PLL_SHORT_ through loose interactions that are not accompanied by demixing is quite interesting from a prebiotic point of view. We could speculate that such an anionic‐ligand‐replacement could provide a diluted ATP (or any other early nucleotide) medium access to preconcentrated pools (through LLPS with MAc) of otherwise diluted oligocations (Figure 7, showing a schematic representation of the phase transitions involving mellitic acid). In other words, MAc‐based droplets could act as recruitment sites for prebiotic oligocations competent to undergo LLPS, as well as various tertiary molecules and inorganic nanoparticles with affinity to this system. For example, we have observed that certain guest organic molecules (e.g., quinacrine) and inorganic microparticles (Fe_2_O_3_·*n*H_2_O) partition into MAc‐PLL droplets (Figure S6). Crucially, due to robust synthesis routes (inorganic oxidation of carbon, etc.), MAc could be formed locally at high concentrations and on short timescales through a variety of abiotic processes. This is why MAc appears to be better suited to overcome high dilution‐related entropic barriers than many other candidate components for early prebiotic protocells involving condensates. Given that the timescales of these different prebiotic processes are difficult to estimate, the high in‐solution thermodynamic stability (including low pH and increased temperatures) of MAc could have been of particular advantage over, for example, hydrolysis‐prone polyphosphates.

## 3 Conclusion

In conclusion, we have identified mellitic acid–the highly stable organic oligoion that forms spontaneously through abiotic mechanisms and, coincidentally, happens to be a reporter molecule of organic matter sought after in astrobiology–as capable of inducing liquid droplets upon interaction with very short oligolysine peptides. Due to the uniquely broad spread of free enthalpies of deprotonation of the acid, mellitate‐polylysine droplets adapt to neutral and moderately acidic environments by “negotiating” the stoichiometry of oligoanion‐oligocation interactions. While the droplets appear to be sufficiently stable under prebiotically relevant temperature and pressure conditions, they are highly dynamic and engage readily in anion‐exchange processes with ATP. We argue that MAc could act as an initial recruiter of diluted cationic molecules to be replaced with other anionic building blocks of early protocells.

## Supporting Information

Experimental section; additional CD, ITC, microscopic and kinetic data on the MAc‐PLL system.

## Conflict of Interest

The authors declare no conflict of interest.

## Supporting information



Supporting Information

## Data Availability

The data that support the findings of this study are available from the corresponding author upon reasonable request.
