# Supplementary material for: Mellitic Acid as a Stable Abiotic Precursor for Liquid‐Liquid Phase Separation: An RNA‐Independent Pathway for Prebiotic Compartmentalization
Source: Chemistry. 2025 Aug 13;31(52):e01526. doi: 10.1002/chem.202501526 (PMC12444742; doi:10.1002/chem.202501526)
Supplement: Supplementary file 1 — Supporting Information [file CHEM-31-e01526-s001.pdf]

Mellitic Acid as a Stable Abiotic Precursor for Liquid-Liquid Phase Separation: An RNA-Independent Pathway for Prebiotic Compartmentalization

Robert Dec[1], Michel W Jaworek[1], Matylda Wacławska[2], Wojciech Dzwolak[2*], Roland Winter[1*]

[1] TU Dortmund University, Department of Chemistry and Chemical Biology, Physical Chemistry I - Biophysical Chemistry, Otto-Hahn Street 4a, 44227 Dortmund, Germany

[2] University of Warsaw, Faculty of Chemistry, Biological and Chemical Research Centre, Pasteur Street 1, 02-093 Warsaw, Poland

* Corresponding authors:

E-mails: wdzwolak@chem.uw.edu.pl, roland.winter@tu-dortmund.de

Supplementary Information

Materials and Methods

Samples

Mellitic acid (MAc), poly-L-lysine (PLL) as hydrobromide salts (PLL_{SHORT} MW 1-5 kDa, PLL_{LONG} 15-30 kDa), adenosine triphosphate (ATP), and apyrase (from potatoes, ATPase ≥ 200 units/mg protein) were purchased from Millipore Sigma (formerly Sigma-Aldrich). Oligo-L-lysine peptides with defined number of ingredient residues (K_5, K_6, K_7, K_8) were custom-synthesized by Biosynth (formerly Pepscan) and delivered as trifluoroacetic acid (TFA) salts. MAc and PLL were dissolved in H_2O at the desired concentrations indicated in the captions while the pH was typically adjusted to 6.5 using diluted NaOH. In the case of pH-dependent LLPS, both NaOH and HCl were used to obtain the desired pH values. Only fresh stock ATP solutions (pH 6.5) were used. As mixing of aqueous MAc, PLL, ATP triggers fast LLPS/precipitation, it was done only on freshly prepared solutions directly before the incubations/measurements.

Optical microscopy

Light microscopy images were recorded on an Eclipse TE2000-U microscope (Nikon Inc.) equipped with a Nikon Plan 20x objective (NA 0.45, WD 7.4) and a Nikon Plan 10x (NA 0.45, WD 4.0) objective. ImageJ software was used for image analysis.

LLPS kinetics

Kinetic optical density (turbidity) measurements at 500 nm were carried out on a UV-1800 Shimadzu UV Spectrophotometer at 30 °C without agitation using a cuvette with 3 mm optical path.

Droplet stability measurements

The stability of MAc-PLL droplets was probed by static light scattering measurement (at 400 nm) on a K2 Multifrequency Phase Fluorometer. For the high-pressure measurements, we used a cuvette with 4 mm pathlength and for the measurements concerning the temperature and ionic strength effect, a 3 mm quartz cuvette. The high-pressure stability was probed at 20 °C whereas temperature stability was probed under atmospheric pressure. In each of these experiments, the response of the MAc-PLL system in terms of light scattering intensity was fast and settled rapidly after each incremental change in these variables. The total elapsed time until the data were acquired at the specified end value applied were: ~ 60 min for salt effect examination (Fig. 4A), 20 min for the pressure scan (Fig. 4B), and 60 min for the temperature scan (Fig. 4C).

Plate reader

Optical density measurements at 500 nm for a series of samples containing sub-stoichiometric amounts of MAc titrated with ATP were carried out with a CLARIOstar plate reader from BMG LABTECH (Offenburg, Germany). The measurements were performed with the use of a 96-well black microplate with transparent bottom. Each well was filled with a 100 µL portion of freshly prepared sample. The reading took place after 5 min of incubation at 25 °C.

Attenuated total reflectance (ATR) FT-IR measurements

The centrifuged high-density phase of the MAc-PLL_{SHORT} system was placed and subsequently dried *in situ* on the diamond surface of the single-reflection ATR accessory of the Nicolet iS50 FT-IR spectrometer from Thermo Fisher Scientific (Waltham, MA, USA) equipped with a DTGS detector. For a single ATR FT-IR spectrum, 32 interferograms of 2 cm^{-1} nominal resolution were co-added. Due to ambiguity in determining real values of the refractive index of the dried droplet material, only uncorrected ATR FT-IR data are shown. In the case of control infrared spectra of non-mixed PLL_{SHORT} and MAc reported at the bottom of Fig. 1B, neat aqueous ~ 1 wt. % solutions of PLL_{SHORT} HBr and MAc (pH-preadjusted to 6.5 with HCl or NaOH) were placed and subsequently dried on the diamond surface of the ATR accessory prior to the recording of the spectra.

Circular dichroism (CD) measurements

For the CD measurement, the dense droplet phase on the MAc-PLL_{SHORT} (pH 6.5, 1:6 MAc:K molar ratio) system was centrifuged and placed in a demountable quartz cuvette of 0.01 mm optical path. The CD spectrum, corrected for the buffer signal, was acquired at room temperature by accumulation of 5 independent spectra (at 200 nm/s scanning rate) on a J-815 S spectropolarimeter from Jasco Corp. (Tokyo, Japan).

Isothermal titration calorimetry (ITC)

The experiments were performed as follows: solutions of PLL_{SHORT} and MAc (pH 7.5) were diluted with pure water to the final concentrations of 250 µM and 300 µM, respectively, and were subsequently degassed. The PLL_{SHORT} solution was transferred to the sample cell and the MAc solution was added to the pipette source cell. The ITC experiments were performed on a MicroCal PEAQ-ITC(Malvern) system at high-feedback mode, reference power 10 µcal s^{-1}, stirring speed of 750 rpm, and 25 °C experimental temperature. A 150 s pre-injection delay was applied for baseline stabilization after equilibration. Subsequently, after a pre-injection delay, 2 µL of MAc weres injected 19 times at 150 s-long intervals. Apparent enthalpy of the reaction,

heat rate (dQ/dt) plots were generated using the 'one set of sites' model within the MicroCal PEAQ-ITC Analysis Software (Malvern).

Additional Figures

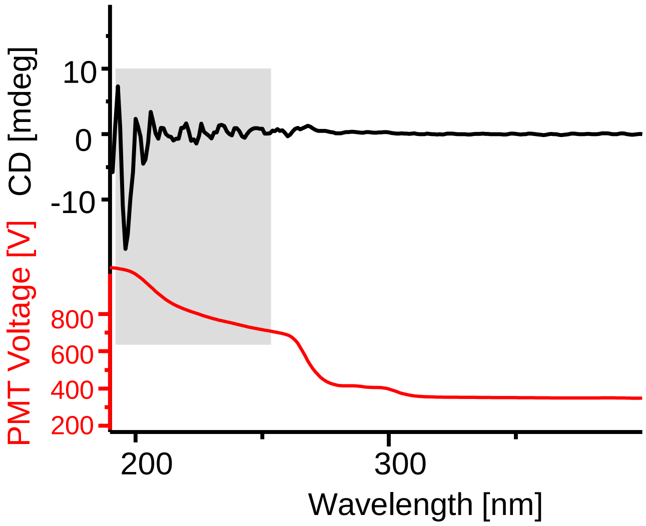

Figure S1. Broad range UV CD spectrum of the centrifuged dense phase of the MAc-PLL$_{SHORT}$ system collected on 0.01 mm optical path (black line). Despite the extremely thin quartz cuvette, the excessive optical density below 250 nm (as reflected by the photomultiplier voltage increasing above 600 V – red line) hampers analysis of the amide CD signal of PLL. There is no evidence of MAc $\pi\pi^*$ transitions (300-250 nm) becoming CD active. Hence, the system remains highly dynamic and fluctuating without higher-order chiral structures involving PLL-bound MAc molecules.

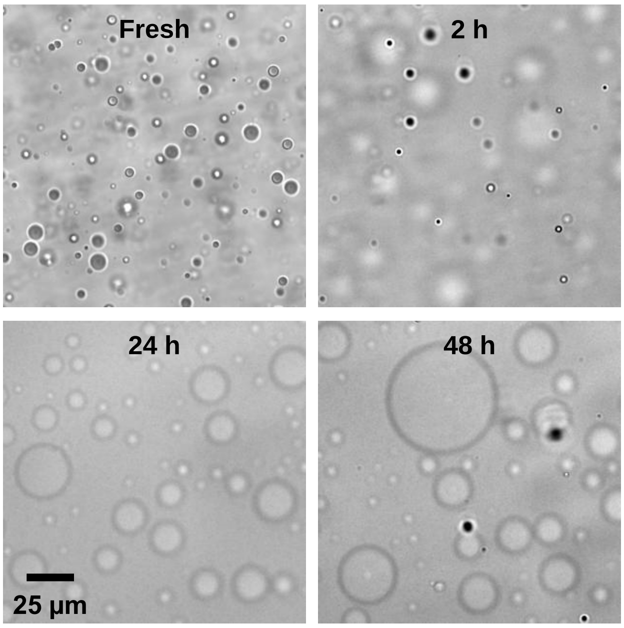

Figure S2. Microscopic probe into the stability of MAc-PLL$_{SHORT}$ droplets over time under ambient conditions. Conditions: PLL$_{SHORT}$ at the concentration of 17.9 mM (per K residue), MAc:K molar ratio 1:6, pH 6.5, room temperature. The scale applies to all images. Fresh

droplets are rather tiny and uniformly distributed throughout the volume of the sample. With the progress of droplet maturation larger entities form and accumulate close to the surface.

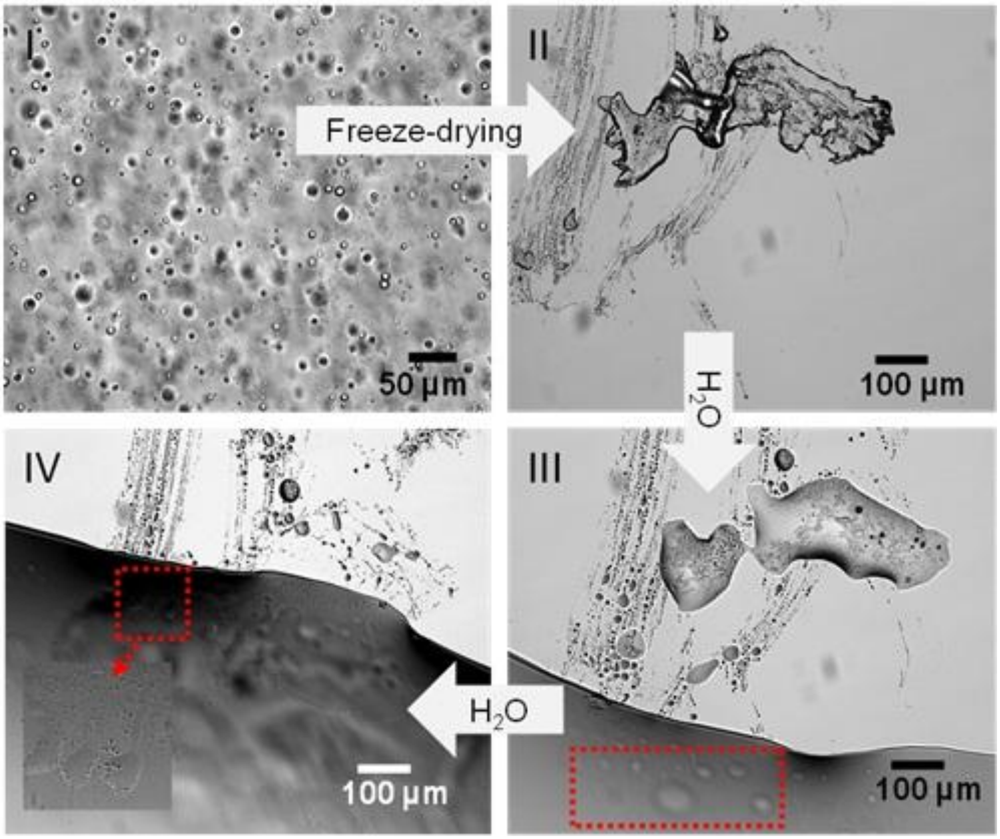

Figure S3. Reconstitution of the liquid droplet state in the MAc-PLL$_{SHORT}$ system subjected to freeze-drying. Liquid droplets of MAc-PLL$_{SHORT}$, pH 6.5, obtained under the typical conditions (I) subjected to freeze-drying (II) and subsequent re-hydration with portions of liquid water (III and IV). Small droplet specimens forming within the diluted liquid phase are highlighted with red frames.

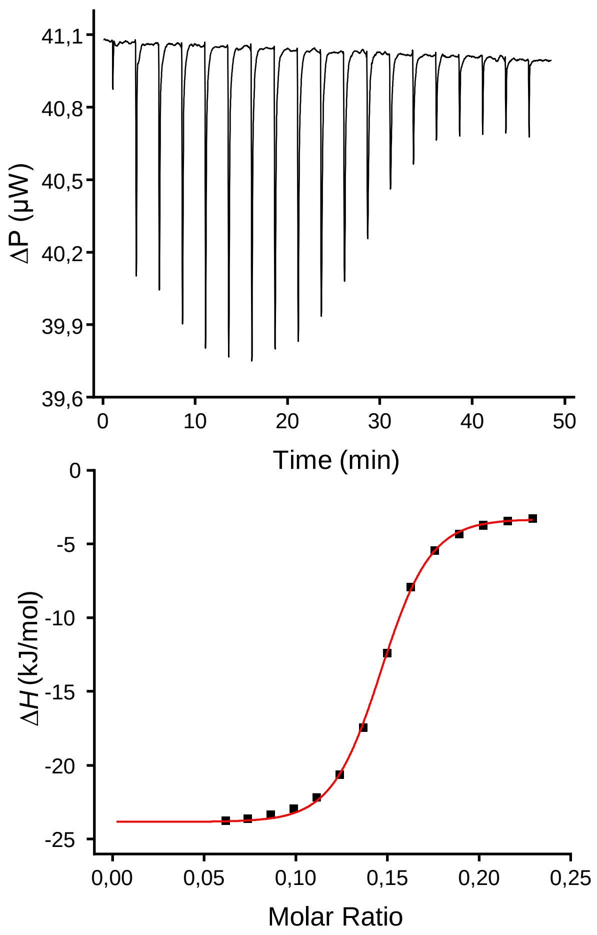

Figure S4. ITC thermogram of MAc solution titrated to aqueous PLL_{SHORT} sample (pH 7.5, 25 ºC) at 150 s-long intervals. The calculated stoichiometric MAc:PLL mixing molar ratio (per K residue) and thermodynamic parameters of binding are: $n = 0.142 \pm 1.2 \cdot 10^{-3}$, $\Delta G = -37.8$ kJ/mol, $\Delta H = -21.4$ kJ/mol, $T\Delta S = 16.4$ kJ/mol.

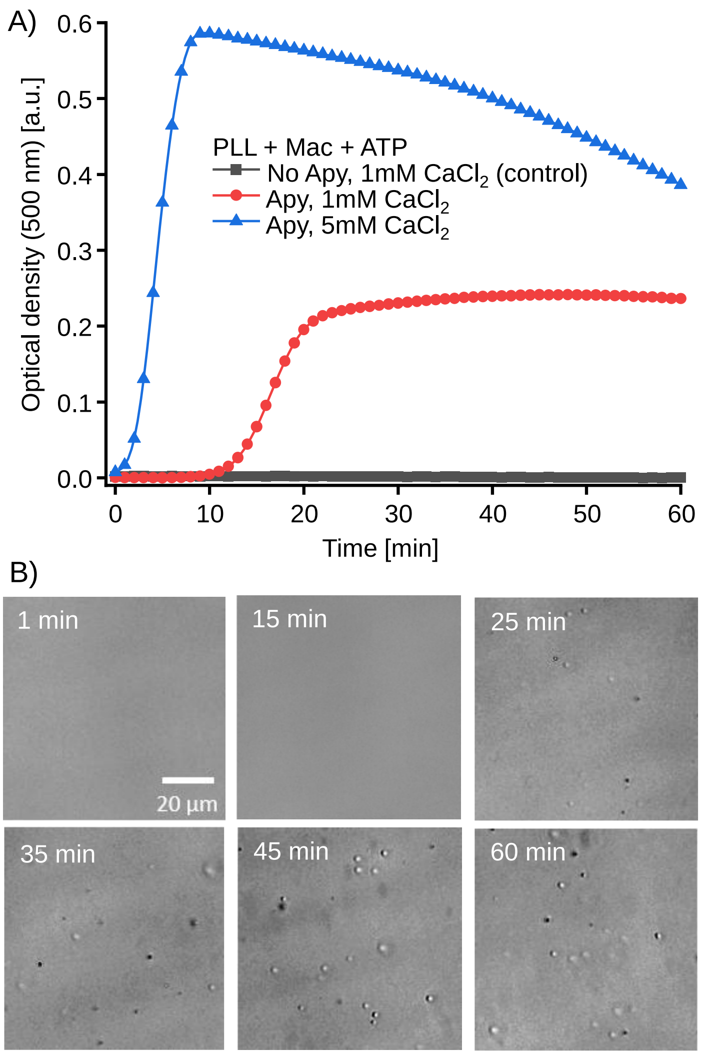

Figure S5. LLPS kinetics of the MAc-PLL$_{SHORT}$-ATP mixture driven by apyrase-induced hydrolysis of ATP probed by optical density at 500 nm (A) and time-lapse optical microscopy (B). Sample conditions: PLL$_{SHORT}$ (17.9 mM per K residue), MAc (1:6 MAc:K molar ratio), ATP (2 ATP molecules per 3 K residue), apyrase (25 (µg/mL), pH 6.5, 30 °C, $CaCl_2$ was present at 1 or 5 mM concentration, as indicated.

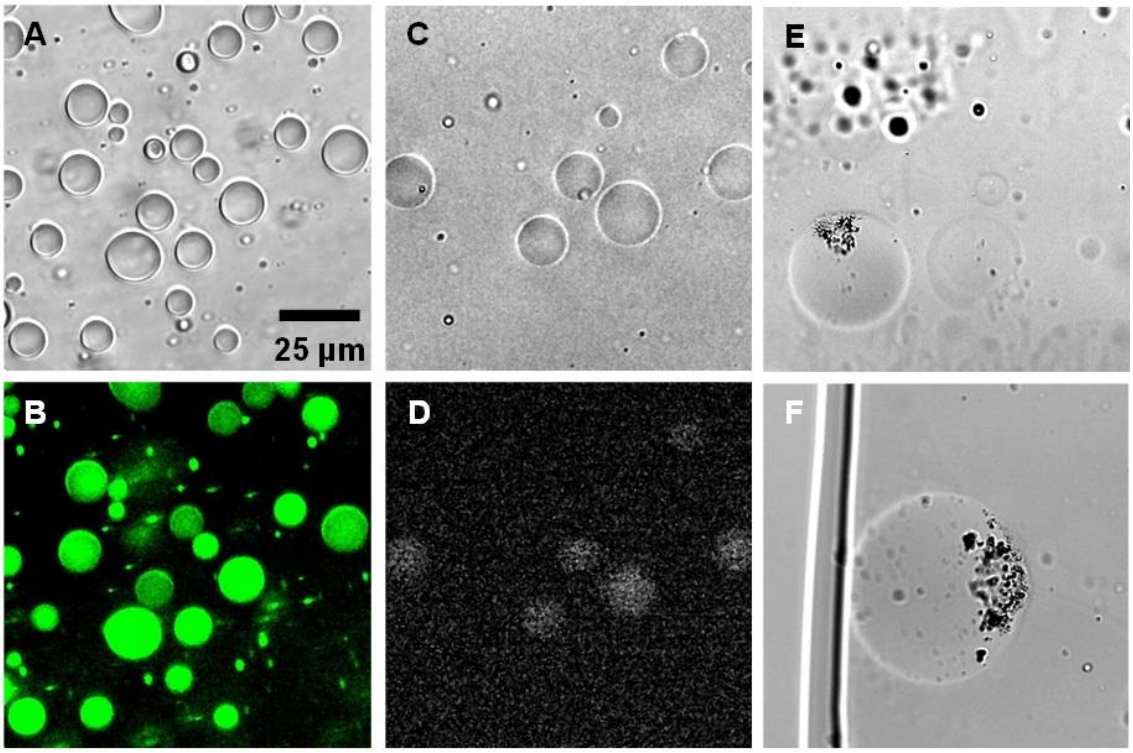

Figure S6. Microscopic brightfield (A, C, E, F) and fluorescence (B, D) images of MAc-PLL_{SHORT} droplets (prepared under the typical conditions of pH and mixing ratio) sequestering various guest macromolecules and microparticles: Alexa 546-labeled poly-D-lysine (4-15 kDa) – A and B (green light excitation); quinacrine – C and D (blue light excitation); sonicated microparticles of $Fe_2O_3 \cdot nH_2O$ - E and F.
